# Beliefs Among Veteran Firearm Owners Regarding Whether Clinicians Should Discuss Firearm Safety With Patients

**DOI:** 10.1001/jamanetworkopen.2023.21219

**Published:** 2023-06-29

**Authors:** Frances M. Aunon, Deborah Azrael, Joseph A. Simonetti, Matthew Miller

**Affiliations:** 1 Veterans Affairs Connecticut Health Care System, West Haven; 2 Department of Psychiatry, Yale School of Medicine, New Haven, Connecticut; 3 Harvard Injury Control Research Center, Harvard T.H. Chan School of Public Health, Boston, Massachusetts; 4 Firearm Injury Prevention Initiative, University of Colorado Anschutz School of Medicine, Aurora; 5 Division of Hospital Medicine, University of Colorado Anschutz School of Medicine, Aurora; 6 Department of Health Sciences, Northeastern University, Boston, Massachusetts

## Abstract

**Importance:**

Veterans are at increased risk of suicide, and guidelines recommend assessing firearm access and counseling to reduce access among patients with elevated suicide risk. How veterans view such discussions is critical to the effectiveness of these interactions.

**Objective:**

To assess whether veteran firearm owners believe clinicians should deliver firearm counseling when patients or their family members are being cared for in specific clinical contexts that suggest heightened risk of firearm injury.

**Design, Setting, and Participants:**

In this cross-sectional study, data were from a probability-based online survey of self-identified veterans who reported owning at least 1 firearm (National Firearms Survey, July 1 to August 31, 2019) and were weighted to generate nationally representative estimates. Data were analyzed from June 2022 to March 2023.

**Main Outcomes and Measures:**

Participants were asked, “As part of routine care, should physicians and/or other health care professionals talk with their patients about firearms and firearm safety if their patient or their patient’s family member (is at risk of suicide; has mental health or behavioral problems; is abusing or addicted to alcohol or drugs; is a victim of domestic violence; has Alzheimer’s disease or another dementia; or is going through a hard time).” Response options included “No,” “Yes, sometimes,” and “Yes, always.” In addition, responses were dichotomized as “Yes, at least sometimes” and “No.”

**Results:**

Of 4030 adults who completed the survey (65% completion rate), 678 (mean [SD] age, 64.7 [13.1] years; 638 [92.9%] male) identified as veteran firearm owners. Across the 6 clinical contexts, support for clinicians “at least sometimes” discussing firearm safety as part of routine care ranged from 73.4% (95% CI, 69.1%-77.3%) when someone is “going through a hard time” to 88.2% (95% CI, 84.8%-90.9%) when someone has “mental health or behavioral problems.” When a patient or family member is at risk for suicide, 79.4% (95% CI, 75.5%-82.8%) of veteran firearm owners responded that clinicians should “at least sometimes” discuss firearms and firearm safety.

**Conclusions and Relevance:**

This study’s findings suggest that most veteran firearm owners believe that clinicians should provide firearm counseling during routine care when a patient or family member is at heightened risk of firearm injury. These findings belie concerns that discussing firearm access with veteran firearm owners is an unacceptable practice.

## Key Points

**Question:**

What proportion of US veterans who own firearms believe that firearm safety counseling should occur in clinical settings when a patient or a patient’s family member is at risk of firearm injury?

**Findings:**

This cross-sectional study of 678 adults found that most veteran firearm owners believed that clinicians should “at least sometimes” discuss firearm safety across all 6 clinical contexts examined (elevated suicide risk, mental health or behavioral problems, drug or alcohol problems, domestic violence, having a hard time, and dementia).

**Meaning:**

These findings suggest that discussing firearm access in clinically indicated situations is viewed as an acceptable routine practice by most veteran firearm owners.

## Teaser

This cross-sectional study assesses the extent to which US veterans who own firearms endorse firearm safety counseling in clinical settings when a patient or a patient’s family member is at risk of firearm injury.

## Introduction

For the past 2 decades, although not prior,^1,2,3,4^ the suicide rate among US veterans has been higher than among age- and sex-matched nonveterans, in part because of higher rates of firearm suicide.^2,5^ In 2019, in response to persistently elevated veteran suicide rates, the US Department of Veterans Affairs (VA) and the US Department of Defense updated their Clinical Practice Guidelines for the Assessment and Management of Patients at Risk for Suicide.^6^ These guidelines included recommendations that clinicians assess firearm access as part of a broader strategy to identify suicide risk factors and discuss ways to reduce firearm access with veterans identified as at heightened risk.

Although the effectiveness of these recommendations on clinical care and veteran suicide is not yet known, the success of these efforts will depend, at minimum, on clinicians’ willingness to discuss firearm-related risk with the approximately 50% of veterans who own firearms.^6^ To date, however, studies in the general US population^7,8^ and among veterans^9^ find that clinician-initiated discussions about firearm safety are uncommon, even among patients who are receiving care for mental health conditions.^10^

The perception that firearm owners may be opposed to discussing firearms has been cited as 1 reason clinicians seldom initiate these conversations.^7,11^ The validity of this perception, however widespread it may be, has been called into question by the few empirical studies^12,13,14,15^ that have sought to examine why clinicians rarely engage in such discussions. Qualitative studies, for example, conducted among veteran and nonveteran samples, suggest that interventions focusing on reducing firearm access are generally considered to be appropriate^13,14^ or appropriate under specific circumstances (eg, delivered by a trusted clinician).^16^ Survey-based research conducted among firearm owners has come to similar conclusions. For example, a recent national study^15^ found that 80% of US adult firearm owners believed that clinicians should “sometimes” or “always” discuss firearm safety with patients when a patient or a patient’s family member is at risk of suicide. To our knowledge, the current study is the first nationally representative survey to assess the extent to which veteran firearm owners endorse firearm safety counseling in clinical settings when a patient or a patient’s family member is at risk of firearm injury.

## Methods

### Design and Study Sample

Data for this cross-sectional study come from the 2019 National Firearms Survey, which was designed by the investigators (M.M. and D.A.) and conducted by the research firm Ipsos from July 1 to August 31, 2019. Race and ethnicity data were included because perceptions about the appropriateness of counseling about firearm safety may differ by race and ethnicity. Race and ethnicity were self-reported at the time respondents joined the panel. Participants were drawn from a nationally representative sample from the 55 000 English-speaking adults (aged ≥18 years) in Ipsos’ probability-based web panel (KnowledgePanel). KnowledgePanel members complete an annual survey assessing firearm ownership and other measures, which allows researchers to draw a subsample using predefined characteristics. Respondents who indicated they lived in a household with firearms were invited to participate in the study. Those who accepted the invitation were then asked additional survey questions, including questions about their veteran status and questions that confirmed both that they lived in a household with firearms and whether they personally owned firearms. Item language and response options are included in eTable 1 in Supplement 1. The study design has been described in detail elsewhere.^17^ For this study, we included firearm owners who self-identified as veterans. Data were deidentified, and the survey was deemed not human participant research under federal guidelines by the institutional review boards at Northeastern University and the Harvard School of Public Health. This study followed the Strengthening the Reporting of Observational Studies in Epidemiology (STROBE) reporting guideline.^18^

### Measures

The primary outcome was veterans’ responses to 6 variations of the survey item: “As part of routine care, should physicians and/or other health care professionals talk with their patients about firearms and firearm safety if their patient or their patient’s family member (is at risk of suicide; has mental health or behavioral problems; is abusing or addicted to alcohol or drugs; is a victim of domestic violence; has Alzheimer’s disease or another dementia; or is going through a hard time).” Response options included “No,” “Yes, sometimes,” and “Yes, always.” We also dichotomized responses as “Yes, at least sometimes” or “No.” Additional items assessed sociodemographic and military service characteristics of respondents and use of Veterans Health Administration (VHA) health care services in the last 12 months (eTable 1 in Supplement 1).

### Statistical Analysis

Data were analyzed from June 2022 to March 2023. Analyses used survey weights provided by Ipsos. These weights account for survey nonresponse and undercoverage or overcoverage imposed by the study-specific sample design. Weights also adjust for benchmark demographic distributions and for population characteristics. For this study, data were weighted to benchmark distributions of firearm-owning households from weighted KnowledgePanel data for sex, age, race/ethnicity, US Census region, metropolitan statistical area status, educational level, household income, and presence of children in the home. We report weighted proportions and corresponding 95% CIs. Analyses were conducted using Stata software, version 16 (StataCorp LLC).

## Results

Of the 4030 adults living in households with firearms who completed the 2019 National Firearms Survey (65% completion rate), 2950 were firearm owners. Of those, 678 veteran firearm owners (mean [SD] age, 64.7 [13.1] years; 638 [92.9%] male and 40 [7.1%] female; 44 [9.3%] Hispanic, 42 [10.1%] non-Hispanic Black, 555 [75.1%] non-Hispanic White, and 37 [5.5%] non-Hispanic other [American Indian or Alaska Native, Asian, Native Hawaiian or Pacific Islander, or more than 1 race]) were included in the analysis. Most respondents were 60 years or older (57.3%; 95% CI, 52.6%-61.9%), 36.5% (95% CI, 32.2%-41.0%) had served in combat, 80.5% (95% CI, 76.2%-84.3%) separated from the military before 2002, and 31.5% (95% CI, 27.5%-35.8%) reported that they had used VHA health care services in the preceding 12 months (Table 1).

**Table 1.** Demographic and Military Service Characteristics of 678 Veteran Firearms Owners, 2019

Characteristic	Unweighted, No.	Weighted, % (95% CI)
Age group, y		
18-29	5	2.3 (0.9-5.9)
30-44	49	10.3 (7.7-13.8)
45-59	160	30.1 (25.8-34.7)
≥60	464	57.3 (52.6-61.9)
Sex		
Male	638	92.9 (90.2-94.9)
Female	40	7.1 (5.1-9.8)
Race and ethnicity		
Hispanic	44	9.3 (6.8-12.6)
Non-Hispanic Black	42	10.1 (7.2-14.1)
Non-Hispanic White	555	75.1 (70.4-79.3)
Non-Hispanic other^a^	37	5.5 (3.5-8.4)
Marital status		
Married or partnered	512	79.5 (76.0-82.7)
Widowed or separated	146	18.0 (15.0-21.4)
Never married	20	2.5 (1.5-4.0)
Children aged >18 y in the household		
No	583	80.7 (76.5-84.3)
Yes	95	19.3 (15.7-23.5)
Educational level		
Less than high school	9	3.6 (1.8-7.1)
High school degree or some college	402	67.0 (62.8-70.9)
Bachelor’s degree or more	267	29.4 (25.8-33.2)
Rurality		
Rural	118	17.1 (14.0-20.8)
Urban	195	28.6 (24.7-32.8)
Suburban	362	54.3 (49.8-58.8)
US region^b^		
Northeast	78	10.5 (8.2-13.2)
Midwest	156	20.8 (17.5-24.6)
South	274	45.6 (41.1-50.1)
West	170	23.1 (19.7-26.9)
Combat veteran		
No	438	63.5 (59.1-67.8)
Yes	237	36.5 (32.2-41.0)
Recent military service (since 2002)		
No	580	80.5 (76.2-84.3)
Yes	96	19.5 (15.7-23.8)

^a^ Non-Hispanic other is composed of respondents who indicated their race as American Indian or Alaska Native, Asian, Native Hawaiian/Pacific Islander, or more than 1 race. Categories were combined due to low individual endorsement.

^b^ Northeast includes Maine, New Hampshire, Vermont, Massachusetts, Rhode Island, Connecticut, New York, Pennsylvania, and New Jersey. Midwest includes North Dakota, South Dakota, Nebraska, Kansas, Minnesota, Iowa, Missouri, Wisconsin, Illinois, Indiana, Michigan, and Ohio. South includes Delaware, Maryland, Washington, DC, Virginia, West Virginia, Kentucky, North Carolina, South Carolina, Georgia, Florida, Tennessee, Alabama, Mississippi, Arkansas, Louisiana, Oklahoma, and Texas. West includes Washington, Oregon, California, Montana, Idaho, Wyoming, Nevada, Utah, Colorado, Arizona, New Mexico, Alaska, and Hawaii.

Across the 6 clinical contexts, support for clinicians “at least sometimes” discussing firearm safety as part of routine care ranged from 73.4% (95% CI, 69.1%-77.3%) when someone is “going through a hard time” to 88.2% (95% CI, 84.8%-90.9%) when someone has “mental health or behavioral problems” (Table 2). When a patient or family member is at risk for suicide, 79.4% (95% CI, 75.5%-82.8%) of veteran firearm owners responded that clinicians should “at least sometimes” discuss firearms and firearm safety.

**Table 2.** Beliefs of 678 Veteran Firearm Owners Regarding Whether Clinicians Should Discuss Firearms and Firearm Safety With Patients Across 6 Clinical Contexts, 2019

Clinical context	Unweighted, No.	Weighted, % (95% CI)
**Is at risk of suicide**		
Yes, at least sometimes	541	79.4 (75.5-82.8)
Yes, always	341	51.9 (47.4-56.4)
Yes, sometimes	200	27.5 (23.8-31.6)
No	130	20.6 (17.3-24.5)
**Has mental health or behavioral problem**		
Yes, at least sometimes	596	88.2 (84.8-90.9)
Yes, always	385	57.3 (52.8-61.7)
Yes, sometimes	211	30.9 (26.9-30.1)
No	72	11.9 (9.2-15.2)
**Is abusing or addicted to alcohol or drugs**		
Yes, at least sometimes	584	85.8 (82.2-88.8)
Yes, always	380	58.0 (53.5-62.3)
Yes, sometimes	204	27.8 (24.2-31.8)
No	84	14.2 (11.2-17.8)
**Is a victim of domestic violence**		
Yes, at least sometimes	574	85.3 (81.7-88.2)
Yes, always	383	57.5 (53.0-61.9)
Yes, sometimes	191	27.8 (24.0-31.9)
No	94	14.7 (11.8-18.3)
**Has Alzheimer disease or another form of dementia**		
Yes, at least sometimes	528	78.3 (74.3-81.8)
Yes, always	313	47.8 (43.3-52.4)
Yes, sometimes	215	30.5 (26.6-34.7)
No	138	21.7 (18.2-25.7)
**Is going through a hard time**		
Yes, at least sometimes	501	73.4 (69.1-77.3)
Yes, always	180	29.1 (25.0-33.5)
Yes, sometimes	321	44.3 (39.9-48.8)
No	166	26.7 (22.7-30.9)

Compared with veteran firearm owners who did not use VHA services within the prior year, those who did were at least as favorably disposed to routine firearm-related conversations in each clinical context (eTable 2 in Supplement 1). Regional differences in how veteran firearm owners responded to our questions were minor; regardless of where firearm owners lived, at least two-thirds endorsed routine lethal means counseling “at least sometimes” (eTable 3 in Supplement 1).

## Discussion

In this nationally representative cross-sectional study, we found that 73% to 88% of veteran firearm owners, including those who received care at the VHA, reported that firearm counseling should be delivered as part of routine care when a patient or someone in their family is at increased risk of firearm injury. These findings are consistent with those from a national survey of firearm owners in which veteran status was not examined^15^ and a geographically diverse sample of veterans receiving mental health treatment.^19^

Our study does not explain why some respondents report that clinicians should never ask about firearms in the clinical contexts we examined (12%-27% depending on the scenario); further work is needed to better understand the perspectives of these individuals. Prior studies^11,13,16,20,21^ suggest that concerns about privacy, the impact of disclosing firearm status on Second Amendment rights, and the potential for discrimination from health care systems and clinicians may be factors that affect the acceptability of clinic-based firearm discussions for some patients.

Our study does not assess how prior experience discussing firearms with clinicians may influence whether these conversations are seen as an acceptable part of routine care. Positive past experiences might favor veterans endorsing these discussions during routine clinical care, whereas negative past experiences might reinforce the opposite. In addition, some firearm owners may have concerns about privacy when disclosing information about firearm ownership or storage to certain clinicians (eg, those they trust less) or in specific systems, such as federal health care systems. However, in our study at least, compared with veteran firearm owners who had not received VHA services in the last year, those who recently received VHA services were at least as likely to endorse firearm counseling.

Most efforts aiming to prevent firearm injuries among adults in clinical spaces have focused on suicide. However, assault-related and unintentional firearm injuries are also important causes of morbidity and mortality in the US, including among veterans. Because the question stem for our primary outcome was not specific to suicide and we assessed scenarios that also place people in the home at heightened risk of firearm assault and unintentional firearm injury, our findings provide support for clinic-based initiatives aiming to prevent injuries from other intents or within other clinical scenarios, such as in dementia care or within the context of intimate partner violence.^22,23^

### Limitations

Our study is subject to limitations. As with any survey, data are self-reported (including veteran status) and thus misclassification is possible, and social desirability or selection biases may have skewed findings. Although residual bias is possible, panel-based surveys have been shown to minimize such biases.^24^

## Conclusions

Most veteran firearm owners in this cross-sectional study agreed that discussing firearms and firearm safety should be an aspect of routine care when someone is at risk of firearm injury. These findings should allay concerns among clinicians, health care leaders, and policy makers that discussing firearms with veterans who are at heightened risk of suicide and other firearm injuries will be viewed as beyond the scope of routine medical care, especially within VHA settings.

## Notes

Supplement 1. **eTable 1.** 2019 National Firearm Survey Items and Response Options **eTable 2.** Veteran Firearm Owners' Beliefs Regarding Whether Clinicians Should Discuss Firearms and Firearm Safety With Patients, by Clinical Context and Use of VHA Healthcare in the Preceding 12 Months, 2019 **eTable 3.** Veteran Firearm Owners' Beliefs Regarding Whether Clinicians Should Discuss Firearms and Firearm Safety With Patients Across Six Clinical Contexts by U.S. Region, 2019

Supplement 2.
Data Sharing Statement
