# Supplementary material for: Beliefs Among Veteran Firearm Owners Regarding Whether Clinicians Should Discuss Firearm Safety With Patients
Source: JAMA Netw Open. 2023 Jun 29;6(6):e2321219. doi: 10.1001/jamanetworkopen.2023.21219 (PMC10311384; doi:10.1001/jamanetworkopen.2023.21219)
Supplement: Supplement 1. — eTable 1. 2019 National Firearm Survey Items and Response Options eTable 2. Veteran Firearm Owners' Beliefs Regarding Whether Clinicians Should Discuss Firearms and Firearm Safety With Patients, by Clinical Context and Use of VHA Healthcare in the Preceding 12 Months, 2019 eTable 3. Veteran Firearm Owners' Beliefs Regarding Whether Clinicians Should Discuss Firearms and Firearm Safety With Patients Across Six Clinical Contexts by U.S. Region, 2019 [file jamanetwopen-e2321219-s001.pdf]

Supplementary Online Content

Aunon FM, Azrael D, Simonetti JA, Miller M. Beliefs among veteran firearm owners regarding whether clinicians should discuss firearm safety with patients. *JAMA Netw Open*. 2023;6(6):e2321219. doi:10.1001/jamanetworkopen.2023.21219

eTable 1. 2019 National Firearm Survey Items and Response Options

eTable 2. Veteran Firearm Owners' Beliefs Regarding Whether Clinicians Should Discuss Firearms and Firearm Safety With Patients, by Clinical Context and Use of VHA Healthcare in the Preceding 12 Months, 2019

eTable 3. Veteran Firearm Owners' Beliefs Regarding Whether Clinicians Should Discuss Firearms and Firearm Safety With Patients Across Six Clinical Contexts by U.S. Region, 2019

This supplementary material has been provided by the authors to give readers additional information about their work.

© 2023 Aunon FM et al. *JAMA Network Open.*

eTable 1. 2019 National Firearm Survey Items and Response Options

Theme	Survey Item	Response Options
Firearm ownership	Do you personally own a gun?	Yes<br>No
Veteran status	Have you ever served on active duty in the U.S. Armed Forces? (Active duty includes serving in the U.S. Armed Forces as well as activation from the Reserves and National Guard)	Yes<br>No
Veterans Health Administration engagement	In the last 12 months have you received any of your health care from the Veterans' Health Administration?	Yes<br>No

© 2023 Aunon FM et al. *JAMA Network Open.*

eTable 2. Veteran Firearm Owners' Beliefs Regarding Whether Clinicians Should Discuss Firearms and Firearm Safety With Patients, by Clinical Context and Use of VHA Healthcare in the Preceding 12 Months, 2019

	Received Veterans Health Administration services in the last 12 months (n=677)					
	Yes			No		
	Unweighted	Weighted		Unweighted	Weighted	
	n	%	95% CI	n	%	95% CI
**All respondents**	216	30.8%	[26.9% to 35.0%]	461	69.1%	[65.0% to 73.1%]
**Is at risk of suicide**						
Yes, at least sometimes	172	78.6%	[71.4% to 84.3%]	369	79.7%	[75.0% to 83.7%]
Yes, always	115	55.4%	[47.5% to 63.0%]	226	50.3%	[44.8% to 55.7%]
Yes, sometimes	57	23.2%	[17.6% to 29.9%]	143	29.5%	[24.8% to 34.6%]
No	42	21.4%	[15.7% to 28.6%]	88	20.3%	[16.3% to 25.0%]
**Has mental health or behavioral problem**						
Yes, at least sometimes	190	90.1%	[84.5% to 93.8%]	406	87.3%	[82.9% to 90.7%]
Yes, always	127	59.1%	[51.1% to 66.6%]	258	56.5%	[51.0% to 61.8%]
Yes, sometimes	63	31.0%	[24.3% to 38.7%]	148	30.8%	[26.1% to 35.9%]
No	22	9.9%	[6.2% to 15.5%]	50	12.7%	[9.4% to 17.1%]
**Is abusing or addicted to alcohol or drugs**						
Yes, at least sometimes	188	88.8%	[82.6% to 92.9%]	396	84.5%	[79.9% to 88.2%]
Yes, always	138	65.4%	[58.6% to 73.3%]	242	54.2%	[48.8% to 59.6%]
Yes, sometimes	50	22.4%	[16.8% to 29.3%]	154	30.3%	[25.7% to 35.2%]
No	23	11.2%	[7.1% to 17.3%]	61	15.5%	[11.8% to 20.1%]
**Is a victim of domestic violence**						
Yes, at least sometimes	185	87.4%	[81.4% to 91.6%]	389	84.3%	[79.7% to 88.0%]
Yes, always	133	64.8%	[57.2% to 71.8%]	250	54.1%	[48.6% to 59.5%]
Yes, sometimes	52	22.5%	[16.9% to 29.4%]	139	30.2%	[25.5% to 35.4%]
No	29	12.7%	[8.4% to 18.6%]	65	15.7%	[12.0% to 20.3%]
**Has Alzheimer's disease or another form of dementia**						
Yes, at least sometimes	172	82.3%	[75.8% to 87.3%]	356	76.5%	[71.4% to 80.1%]
Yes, always	108	53.4%	[45.5% to 61.2%]	205	45.3%	[39.9% to 50.8%]

© 2023 Aunon FM et al. *JAMA Network Open.*

Yes, sometimes	64	28.8%	[22.4% to 36.3%]	151	31.2%	[26.5% to 36.4%]
No	39	17.7%	[12.7% to 24.2%]	99	23.5%	[19.1% to 28.6%]
**Is going through a hard time**						
Yes, at least sometimes	169	80.0%	[69.1% to 77.3%]	332	70.4%	[64.9% to 75.3%]
Yes, always	77	38.0%	[30.5% to 46.1%]	103	25.0%	[20.3% to 30.4%]
Yes, sometimes	92	42.0%	[34.3% to 50.1%]	229	45.4%	[40.1% to 50.8%]
No	42	20.0%	[14.5% to 27.0%]	124	29.6%	[24.7% to 35.1%]
Abbreviations: CI% to confidence interval						

© 2023 Aunon FM et al. *JAMA Network Open.*

eTable 3. Veteran Firearm Owners' Beliefs Regarding Whether Clinicians Should Discuss Firearms and Firearm Safety With Patients Across Six Clinical Contexts by U.S. Region, 2019

	**Northeast** (n=76)			**Midwest** (n=153)			**South** (n=273)		
	**Unweighted N**	**Weighted % 95% CI**		**Unweighted N**	**Weighted % 95% CI**		**Unweighted N**	**Weighted % 95% CI**	
**Is at risk of suicide**									
Yes, at least sometimes	57	71.8%	[58.4% to 82.2%]	128	82.0%	[72.9% to 88.6%]	224	82.5%	[76.8% to 87.0%
Yes, always	37	41.5%	[29.9% to 54.1%]	84	56.9%	[47.3% to 65.9%]	142	55.7%	[48.6% to 62.6%
Yes, sometimes	20	30.3%	[19.8% to 43.4%]	44	25.2%	[18.4% to 33.4%]	82	26.7%	[21.1% to 33.2%
No	19	28.2%	[17.8% to 41.6%]	25	18.0%	[11.4% to 27.1%]	49	17.6%	[13.0% to 23.2%
**Has mental health or behavioral problem**									
Yes, at least sometimes	62	82.6%	[71.1% to 90.1%]	138	89.5%	[80.9% to 94.5%]	247	90.6%	[85.7% to 93.9%
Yes, always	40	51.0%	[38.4% to 63.5%]	98	64.8%	[55.2% to 73.3%]	157	59.0%	[51.9% to 65.7%
Yes, sometimes	22	31.5%	[20.9% to 44.6%]	40	24.7%	[17.7% to 33.3%]	90	31.6%	[25.5% to 38.3%
No	14	17.4%	[9.9% to 29.0%]	13	10.5%	[5.5% to 19.2%]	25	9.5%	[6.2% to 14.3%]
**Is abusing or addicted to alcohol or drugs**									
Yes, at least sometimes	60	80.3%	[68.4% to 88.5%]	134	86.2%	[76.8% to 92.1%]	242	87.9%	[82.7% to 91.7%
Yes, always	36	45.3%	[33.0% to 58.1%]	98	64.3%	[54.7% to 72.9%]	157	61.1%	[54.1% to 67.6%
Yes, sometimes	24	35.1%	[23.8% to 48.4%]	36	21.9%	[15.5% to 29.9%]	85	26.9%	[21.4% to 33.1%
No	15	19.7%	[11.5% to 31.6%]	17	13.8%	[7.9% to 23.2%]	31	12.1%	[8.3% to 17.3%]
**Is a victim of domestic violence**									
Yes, at least sometimes	60	79.6%	[67.6% to 87.9%]	128	81.6%	[72.2% to 88.3%]	240	89.0%	[84.0% to 92.6%
Yes, always	43	53.4%	[40.7% to 65.7%]	92	59.9%	[50.2% to 68.8%]	167	62.8%	[55.7% to 69.4%
Yes, sometimes	17	26.2%	[16.3% to 39.3%]	36	21.8%	[15.3% to 30.1%]	73	26.2%	[20.5% to 32.9%
No	16	20.4%	[12.1% to 32.4%]	24	18.4%	[11.7% to 27.8%]	31	11.0%	[7.4% to 16.0%]
**Has Alzheimer's disease or another form of dementia**									
Yes, at least sometimes	53	67.6%	[54.6% to 78.3%]	124	79.0%	[69.3% to 86.3%]	212	79.8%	[73.8% to 84.7%
Yes, always	28	31.6%	[21.4% to 44.0%]	81	54.4%	[44.7% to 63.7%]	130	51.1%	[43.9% to 58.3%
Yes, sometimes	25	35.9%	[24.6% to 49.2%]	43	24.7%	[17.9% to 33.0%]	82	28.7%	[22.8% to 35.5%
No	23	32.4%	[21.7% to 45.4%]	27	21.0%	[13.7% to 30.7%]	58	20.2%	[15.3% to 26.2%
**Is going through hard time**									
Yes, at least sometimes	53	69.2%	[56.1% to 79.8%]	125	80.6%	[71.4% to 87.4%]	200	73.1%	[66.2% to 79.0%
Yes, always	16	15.9%	[9.3% to 25.8%]	58	40.4%	[31.2% to 50.2%]	68	30.7%	[24.1% to 38.2%
Yes, sometimes	37	53.4%	[40.6% to 65.7%]	67	40.3%	[31.6% to 49.7%]	132	42.4%	[35.5% to 49.5%
No	22	30.8%	[20.2% to 43.9%]	27	19.4%	[12.6% to 28.6%]	71	26.9%	[21.0% to 33.8%

Note: Northeast includes ME, NH, VT, MA, RI, CT, NY, PA, NJ; Midwest includes ND, SD, NE, KS, MN, IA, MO, WI, IL, IN, MI, OH; South includes DE, MD, DC, VA, WV, K
TX; West includes WA, OR, CA, MT, ID, WY, NV, UT, CO, AZ, NM, AK, HI.

Abbreviations: CI% to confidence interval

© 2023 Aunon FM et al. *JAMA Network Open.*
