# Supplementary material for: Beliefs Among Veteran Firearm Owners Regarding Whether Clinicians Should Discuss Firearm Safety With Patients
Source: JAMA Netw Open. 2023 Jun 29;6(6):e2321219. doi: 10.1001/jamanetworkopen.2023.21219 (PMC10311384; doi:10.1001/jamanetworkopen.2023.21219)
Supplement: Supplement 2. — Data Sharing Statement [file jamanetwopen-e2321219-s002.pdf]

Data Sharing Statement

Aunon. Beliefs Among Veteran Firearm Owners Regarding Whether Clinicians Should Discuss Firearm Safety With Patients. *JAMA Netw Open*. Published June 29, 2023. doi:10.1001/jamanetworkopen.2023.21219

Data
Data available: No

Additional Information
Explanation for why data not available: Statistical code is available by request from Dr. Aunon; data is accessible without investigator support through Ipsos (KnowledgePanel) https://www.ipsos.com/en-us/en-us/contact
